# Could α-Synuclein Modulation of Insulin and Dopamine Identify a Novel Link Between Parkinson’s Disease and Diabetes as Well as Potential Therapies?

**DOI:** 10.3389/fnmol.2018.00465

**Published:** 2018-12-21

**Authors:** Guadalupe Vidal-Martinez, Barbara Yang, Javier Vargas-Medrano, Ruth G. Perez

**Affiliations:** Department of Biomedical Sciences, Center of Emphasis in Neurosciences, Graduate School of Biomedical Sciences, Paul L. Foster School of Medicine, Texas Tech University Health Sciences Center El Paso, El Paso, TX, United States

**Keywords:** alpha-synuclein, dopamine, insulin, Kir6.2, LAG3, Parkinson’s disease, type 2 diabetes

## Abstract

Characterizing the normal function(s) of the protein α-Synuclein (aSyn) has the potential to illuminate links between Parkinson’s disease (PD) and diabetes and also point the way toward new therapies for these disorders. Here we provide a perspective for consideration based on our discovery that aSyn normally acts to inhibit insulin secretion from pancreatic β-cells by interacting with the Kir6.2 subunit of the ATP-sensitive potassium channel (K-ATP). It is also known that K-ATP channels act to inhibit brain dopamine secretion, and we have also shown that aSyn is a normal inhibitor of dopamine synthesis. The finding, that aSyn modulates Kir6.2 and other proteins involved in dopamine and insulin secretion, suggests that aSyn interacting proteins may be negatively impacted when aSyn aggregates inside cells, whether in brain or pancreas. Furthermore, identifying therapies for PD that can counteract dysfunction found in diabetes, would be highly beneficial. One such compound may be the multiple sclerosis drug, FTY720, which like aSyn can stimulate the activity of the catalytic subunit of protein phosphatase 2A (PP2Ac) as well as insulin secretion. In aging aSyn transgenic mice given long term oral FTY720, the mice had reduced aSyn pathology and increased levels of the protective molecule, brain derived neurotrophic factor (BDNF) (Vidal-Martinez et al., 2016). In collaboration with medicinal chemists, we made two non-immunosuppressive FTY720s that also enhance PP2Ac activity, and BDNF expression (Vargas-Medrano et al., 2014; Enoru et al., 2016; Segura-Ulate et al., 2017a). FTY720 and our novel FTY720-based-derivatives, may thus have therapeutic potential for both diabetes and PD.

We and others have shown that aSyn protein has important normal functions that are associated with its ability to interact with other molecules in a chaperone-like manner (Jenco et al., 1998; Jensen et al., 1999; Ostrerova et al., 1999; Jo et al., 2000; Murphy et al., 2000; Souza et al., 2000; Hashimoto et al., 2002; Seo et al., 2002; Kim et al., 2004; Acosta-Martinez and Levine, 2007; Martinez et al., 2007; Klegeris et al., 2008; Gorbatyuk et al., 2010b; Aoki and Li, 2011; Jin et al., 2011; Oaks and Sidhu, 2011; Bartels et al., 2014; Lautenschläger et al., 2018). Over the years, our laboratory has identified several aSyn-interacting proteins and organelles. These include: tyrosine hydroxylase, also called tyrosine 3-monooxygenase (EC 1.14.16.2) (TH), the rate limiting dopamine biosynthetic enzyme that localizes on vesicles and mitochondria with aSyn (Perez et al., 2002; Jin et al., 2007; Alerte et al., 2008); the next enzyme in the dopamine biosynthetic pathway, aromatic amino acid decarboxylase, AADC, also called dopa decarboxylase (Tehranian et al., 2006); the catalytic subunit of protein phosphatase 2A (PP2Ac) (Peng et al., 2005; Lou et al., 2010); and the 14-3-3ζ adapter protein, which also localize to mitochondria to help regulate dopamine synthesis at that organelle (Wang et al., 2009).

With normal aSyn function(s) in mind and knowing that aSyn normally interacts with and regulates many other molecules, we long ago hypothesized that a loss of aSyn function could be especially detrimental to dopaminergic neurons in a manner to contribute to nigral PD pathology (Perez and Hastings, 2004; Porras and Perez, 2014). We have tested this hypothesis in multiple models over the years. These include using aSyn lentivirus in mice, brains from familial PD and Dementia with Lewy Bodies (DLB) subjects, cell free assays, and aSyn transgenic mice where we confirmed that TH and PP2A activities become dysregulated when aSyn aggregates (Alerte et al., 2008; Wu et al., 2012; Farrell et al., 2014). This demonstrates an important normal role for soluble aSyn in the regulation of key aSyn-interacting molecules. Others have also shown that sustaining normal aSyn levels contributes significantly to neuronal viability, further solidifying a major role for soluble aSyn in optimal brain health (Gorbatyuk et al., 2010a; Kanaan and Manfredsson, 2012; Benskey et al., 2016; Collier et al., 2016).

A lesser known function of aSyn is our discovery that the protein is highly expressed in pancreatic beta cells where it interacts with Kir6.2 on insulin secretory granules, acting to downregulate insulin secretion (Geng et al., 2011). In data from co-immunoprecipitation experiments we show that aSyn and Kir6.2 interact with each other in the pancreas and in islet cell cultures, as can be appreciated in Figure 1. The methods used for these experiments are detailed in our figure legend. In this same paper, striking immunohistochemical images generated by Drs. Geng and Drain confirm near perfect overlapping localization of aSyn not only with Kir6.2, but also with Sur1, Insulin, and C peptide in beta cells (Geng et al., 2011).

**FIGURE 1:**
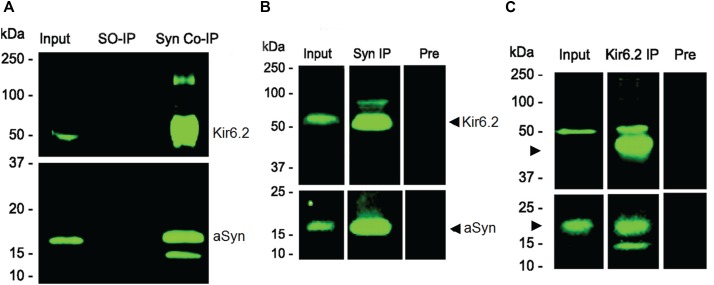
Co-immunoprecipitation (Co-IP) of Kir6.2 with aSyn from pancreas **(A)** and from pancreatic islet cell cultures **(B,C)**. **(A)** Anti-aSyn antibody (BD Biosciences, Cat # BD610787) was used to immunoprecipitate aSyn protein from mouse pancreatic tissue extracts. Immunocomplexes are characterized on immunoblots using anti-Kir6.2 (Santa Cruz Biotechnology, Cat # sc-11228) and anti-aSyn (Santa Cruz Biotechnology, Cat # sc-7011-R) antibodies. Equivalent aliquots of the initial input of each extract (Input) were analyzed. Homogenates in which secondary antibody only was used (SO-IP) served as a negative control. Both aSyn and Kir6.2 were co-immunoprecipitated with the anti-aSyn antibody (Syn Co-IP). **(B)** Binding interactions between Kir6.2 and aSyn are also seen in a representative Co-IP experiment using mouse islet cells grown in culture. Immunoblots were reacted with anti-aSyn antibody (Santa Cruz Biotechnology, Cat # sc-7011-R; in **B** and **C**, bottom panel) or anti-Kir6.2 antibody (Santa Cruz Biotechnology, Cat # sc-20809, H55; in **B** and **C**, top panel). Both Kir6.2 and aSyn are present on immunoblots in initial homogenates (Input), and were enriched after anti-aSyn antibody Co-IP (Syn-1, BD 610787; Syn IP). Specificity was confirmed using pre-adsorbed Syn-1 antibody (Pre), which efficiently reduced levels of protein that were co-immunoprecipitated. (**C**) Binding interactions between Kir6.2 and aSyn in a representative experiment using mouse islet cells also show the presence of Kir6.2 and aSyn in the initial homogenate (Input) as well as in the Co-IP performed using the anti-Kir6.2 antibody (Santa Cruz Biotechnology, Cat # sc-20809, H55; Kir6.2 IP), with specificity demonstrated in a Co-IP using pre-immune serum + beads (Pre). Molecular weights, determined from pre-stained standards, are shown on the left. Data from Geng et al. (2011) reprinted with permission obtained from the Copyright Clearance Center.

The aSyn/Kir6.2 interaction becomes more intriguing because in brain, neuronal Kir6.2 is found in axons and dendrites (Patel et al., 2011; Trimmer, 2015) where it plays an active role in the downregulation of dopamine secretion (Avshalumov and Rice, 2003; Bao et al., 2005; Shi et al., 2008; Patel et al., 2011; Trimmer, 2015). It remains unknown if Kir6.2 and aSyn interact and colocalize on neurotransmitter secretory vesicles in a manner to downregulate dopamine secretion similar to its effects on Kir6.2 in insulin secretory granules. Still, this possibility and other cumulative findings lead us to propose that a loss of aSyn/Kir6.2 interactions that may occur when aSyn aggregates could produce over-secretion of insulin and dopamine, although this remains largely unexplored. This possibility has further implications because there are multiple emerging lines of evidence supporting links between type 2 diabetes mellitus (T2DM) and PD comorbidity (Hu et al., 2007; Driver et al., 2008; Cereda et al., 2011, 2013; Palacios et al., 2011; Schernhammer et al., 2011; Kotagal et al., 2012; Aviles-Olmos et al., 2013; Santiago and Potashkin, 2013; Marcelo et al., 2014; Zhang and Tian, 2014; Santiago et al., 2017; Foltynie et al., 2018), as has been recently been confirmed (De Pablo-Fernandez et al., 2018).

In this regard, protein misfolding and insulin resistance are common to both T2DM and PD (Athauda and Foltynie, 2016). In diabetes, this protein misfolding implicates the islet amyloid polypeptide protein (IAPP, also known as amylin), which is a short peptide that is packaged and secreted along with insulin from pancreatic beta cells (Moore and Cooper, 1991). IAPP/amylin plays a role in glycemic regulation and is known to adopt abnormal conformations that can permeabilize synthetic vesicles in a pore-like manner akin to findings for aSyn protein (Anguiano et al., 2002). This has led some to propose that IAPP/amylin oligomers may act in a prion-like manner in the pancreatic islet cells of diabetics to spur disease onset and/or progression, as some data tend to support (Mukherjee et al., 2015, 2017). In addition, cross-seeding of aSyn and IAPP/amylin has been shown to accelerate the aggregation of both of these aggregation prone proteins (Horvath and Wittung-Stafshede, 2016), raising the possibility that aSyn may accumulate among the amyloids in pancreatic beta cells. This was recently confirmed in pancreatic tissues from subjects with synucleinopathies (Martinez-Valbuena et al., 2018). In addition, there is evidence that in nigral dopamine neurons of individuals with idiopathic/sporadic PD, there is a dysregulation of miR-126, a microRNA involved in the regulation of insulin/IGF-1/phosphatidylinositol-3-kinase (PI3K)/AKT and extracellular signal-regulated kinase (ERK) signaling (Kim et al., 2014; Briggs et al., 2015). Further, it is well-appreciated that insulin signaling contributes significantly to normal brain function and becomes dysregulated in neurodegeneration (Bomfim et al., 2012; Bamji-Mirza et al., 2014; Gao et al., 2015). Together these findings provide strong support for an association between T2DM and PD in which aSyn may play a pivotal role.

It is well-established that aSyn misfolding contributes to PD as well as to other synucleinopathies, such as DLB and multiple system atrophy (MSA) (Galvin et al., 2001; Goedert, 2001; Trojanowski and Lee, 2003). It has further been shown that aSyn oligomerization, to form preformed fibrils (PFF), can induce a prion-like spread of aSyn and cell death in PD models (Volpicelli-Daley et al., 2011, 2014; Dryanovski et al., 2013; Polinski et al., 2018). Also, aSyn PFF uptake *in vitro* and *in vivo* is modulated specifically by the LAG3 receptor, which has been shown to contribute to pathological aSyn transmission (Mao et al., 2016). Moreover, LAG3 has also been implicated in autoimmune diabetes (Bettini et al., 2011; Zhang et al., 2017), providing further evidence for potential overlap between diabetes and PD.

It is also becoming accepted that aSyn plays a role in inducing innate and adaptive immunity in PD (Allen Reish and Standaert, 2015), arising, at least in part, by aSyn activating microglial cells, which stimulates neuroimmunity (Sanchez-Guajardo et al., 2013). A role for aSyn in metabolism has also been reported in the Thy1 promoter parkinsonian A53T mice, where aSyn pathology was found to drive metabolic abnormalities in that PD model (Rothman et al., 2014). Inflammation and activated innate immunity have been shown to play a role in the pathogenesis of T2DM (Pickup, 2004) and inflammation is known to be common in diabetes and other metabolic disorders (Hotamisligil et al., 1993; Zhong et al., 2017). Based on these findings, it thus would be prudent to evaluate parkinsonian mouse models for potential overlapping pathology related to PD and T2DM.

First described by James Parkinson in the early 1800s, it is remarkable to find that in his initial description of the disorder that was later named after him, he was among the first to suggest that the “shaking palsy” may be caused by “compression of the brain, or dependent on *partial exhaustion of the energy of that organ*” (Parkinson, 2002). This suggests that Parkinson himself had anticipated a potential role for metabolic dysregulation in brain as contributing to the disease pathology. Yet, even 200 years later the scientific community continues to search to identify the cause for PD and for successful therapies that will counteract PD pathology.

In our search to identify protective therapies for PD, we began studying FTY720 (fingolimod, Gilenya), a Food and Drug Administration approved therapy for the demyelinating brain disorder, multiple sclerosis (Brinkmann et al., 2010). We first evaluated FTY720 based on its ability to stimulate PP2A activity (Oaks et al., 2013; Vargas-Medrano et al., 2014). This is because our research had revealed that aSyn is a normal stimulator of PP2A catalytic subunit activity (Peng et al., 2005), and that PP2A activity is significantly diminished *in vivo* if aSyn becomes insoluble and accumulates in Lewy bodies (Wu et al., 2012; Farrell et al., 2014). Later, others showed that FTY720 stimulates the expression of the protective molecule BDNF *in vitro* and *in vivo* (Deogracias et al., 2012). Thus, we began testing FTY720 in aging parkinsonian aSyn A53T transgenic mice and found that the mice not only tolerate long term FTY720 treatment, but also have behavioral improvement, increased BDNF expression, and reduced Lewy body-like aSyn pathology when compared to transgenic littermates treated with a vehicle control solution (Vidal-Martinez et al., 2016). In control experiments Vidal-Martinez et al. (2016) also show that blocking BDNF signaling accelerates aSyn aggregation that is reversed by co-delivering FTY720 with the TrkB blocker, ANA-12. Moreover, in addition to being able to improve both glial and neuronal cell functions (Balatoni et al., 2007; Miron et al., 2008; Kim et al., 2011; Gao et al., 2012; Vargas-Medrano et al., 2014; Cipriani et al., 2015; Segura-Ulate et al., 2017b), FTY720 has been shown to have potent anti-diabetic activity including an ability to stimulate insulin secretion (Fu et al., 2001; Yang et al., 2003; Kendall and Hupfeld, 2008; Zhao et al., 2012; Moon et al., 2013). Remarkably, insulin itself can stimulate dopamine release (Stouffer et al., 2015; Sulzer et al., 2016), confirming related effects on insulin and dopamine in brain and pancreas that are highly relevant to PD and T2DM. In addition, there is compelling evidence that dopamine itself is produced within beta cells of the human pancreas, where it becomes packaged along with insulin and acts to negatively regulate insulin secretion (Simpson et al., 2012). Future studies will be required to determine if aSyn binding to Kir6.2 occurs in brain to modulate dopamine similarly to its effects on insulin release. Additional studies to assess potential benefits of our novel FTY720-derivative compounds in pancreatic beta cells and neurons are also required. Cumulatively, the findings concerning the comorbidity of diabetes with PD, and the overlapping interactions between aSyn and key regulatory molecules in brain and pancreas open the door to further explore potential novel therapies that may benefit both disorders that affect a large percentage of our rapidly aging population, worldwide.

## Author Contributions

All authors contributed to literature searches, reading, writing, and/or editing of this manuscript. The review was conceived of by RP who also obtained permission to reprint data via Copyright Clearance Center for Figure 1. BY and JV-M provided intellectual content at all stages. After review, recommendations led us to seek the expertise of GV-M to further improve writing and content.

## Conflict of Interest Statement

RP has filed a patent for FTY720 derivative compounds. Compositions and Methods for the Treatment of Parkinson’s Disease. Publication# 2015/0290145.

The remaining authors declare that the research was conducted in the absence of any commercial or financial relationships that could be construed as a potential conflict of interest.
